# Flexible growing rods: a pilot study to determine if polymer rod constructs may provide stability to skeletally immature spines

**DOI:** 10.1186/1748-7161-10-S2-S16

**Published:** 2015-02-11

**Authors:** Donita I Bylski-Austrow, David L Glos, Anne C Bonifas, Max F Carvalho, Matthew T Coombs, Peter F Sturm

**Affiliations:** 1 Orthopaedics, Cincinnati Children's Hospital Medical Center, Cincinnati, OH, 45229-3030 USA; 2 University of Cincinnati, Cincinnati, OH, USA

## Abstract

**Background:**

Surgical treatments for early onset scoliosis (EOS), including growing rod constructs, involve many complications. Some are due to biomechanical factors. A construct that is more flexible than current instrumentation systems may reduce complications. The purpose of this preliminary study was to determine spine range of motion (ROM) after implantation of simulated growing rod constructs with a range of clinically relevant structural properties. The hypothesis was that ROM of spines instrumented with polyetheretherketone (PEEK) rods would be greater than metal rods and lower than noninstrumented controls. Further, adjacent segment motion was expected to be lower with polymer rods compared to conventional systems.

**Methods:**

Biomechanical tests were conducted on 6 skeletally immature porcine thoracic spines (domestic swine, 35-40 kg). Spines were harvested after death from swine that had been utilized for other studies (IACUC approved) which had not involved the spine. Paired pedicle screws were used as anchors at proximal and distal levels. Specimens were tested under the following conditions: control, then dual rods of PEEK (6.25 mm), titanium (4 mm), and CoCr (5 mm) alloy. Lateral bending (LB) and flexion-extension (FE) moments of ±5 Nm were applied. Vertebral rotations were measured using video. Differences were determined by two-tailed t-tests and Bonferroni correction with four primary comparisons: PEEK vs control and PEEK vs CoCr, in LB and FE (α=0.05/4).

**Results:**

In LB, ROM of specimens with PEEK rods was lower than control at each instrumented level. ROM was greater for PEEK rods than both Ti and CoCr at every instrumented level. Mean ROM at proximal and distal noninstrumented levels was lower for PEEK than for Ti and CoCr. In FE, mean ROM at proximal and distal noninstrumented levels was lower for PEEK than for metal. Combining treated levels, in LB, ROM for PEEK rods was 35% of control (p<0.0001) and 270% of CoCr rods (p<0.01). In FE, ROM with PEEK was 27% of control (p<0.001) and 180% of CoCr (p<0.01).

**Conclusions:**

PEEK rods decreased flexibility versus noninstumented controls, and increased flexibility versus metal rods. Smaller increases in ROM at proximal and distal adjacent motion segments occurred with PEEK compared to metal rods, which may help decrease junctional kyphosis. Flexible growing rods may eventually help improve treatment options for young patients with severe deformity.

## Background

Early onset scoliosis (EOS), which first presents in children under the age of 5 years, has high morbidity and mortality rates due to chest wall deformities that restrict pulmonary development. Current treatment methods include casting, bracing, rib expansion and spine distraction. Conservative treatments such as bracing or casting are not always effective. Surgical treatments such as rib expansion and growing rod instrumentation typically require multiple surgeries and involve many complications. Most growing rods are lengthened at 6 month intervals [1]. Complications include infection, rod breakage, screw pull-out, joint fusion, and junctional kyphosis. Constructs that lengthen magnetically are under investigation. Although these would reduce repeated surgeries, they are relatively stiff and bulky, the elongating section cannot be contoured, and MRI is contraindicated. Physicians have suggested that a more flexible growing rod construct might result in a more flexible spine with fewer surgical complications, provided construct strength is sufficient for curve correction.

Polymer rods have been previously investigated for use in adult, short segment, lumbar spine surgery [2,3]. The material, polyetheretherketone (PEEK), has a lower modulus than traditional rod materials, which might allow for greater range of motion (ROM) than the current metal rods of cobalt-chrome (CoCr) or titanium (Ti) alloys. However, it is not known whether PEEK rods of the required length and diameter have sufficient stability to withstand physiological loads and provide distraction and curve correction, even in very young children with low physiological demands. The purpose of this study was to determine changes to the biomechanical properties of skeletally immature spines after implantation of simulated growing rod constructs with a range of clinically relevant structural properties. The hypothesis was that ROM of spines instrumented with PEEK rods would be both much greater than metal rods and significantly lower than noninstrumented controls. It was expected that ROM with PEEK rods would remain closer to controls than to metal rod constructs, and so be unlikely to provide sufficient stability. Further, adjacent segment motion was expected to be lower with polymer rods compared to conventional systems.

## Methods

*In vitro* biomechanical tests were conducted on six skeletally immature porcine thoracic spines (domestic pigs, 10-14 weeks of age, body mass 35-40 kg). Spines were harvested after death of swine that had been utilized for other studies (approved by IACUC, University of Cincinnati) which had not involved the spine.

**Figure 1:**
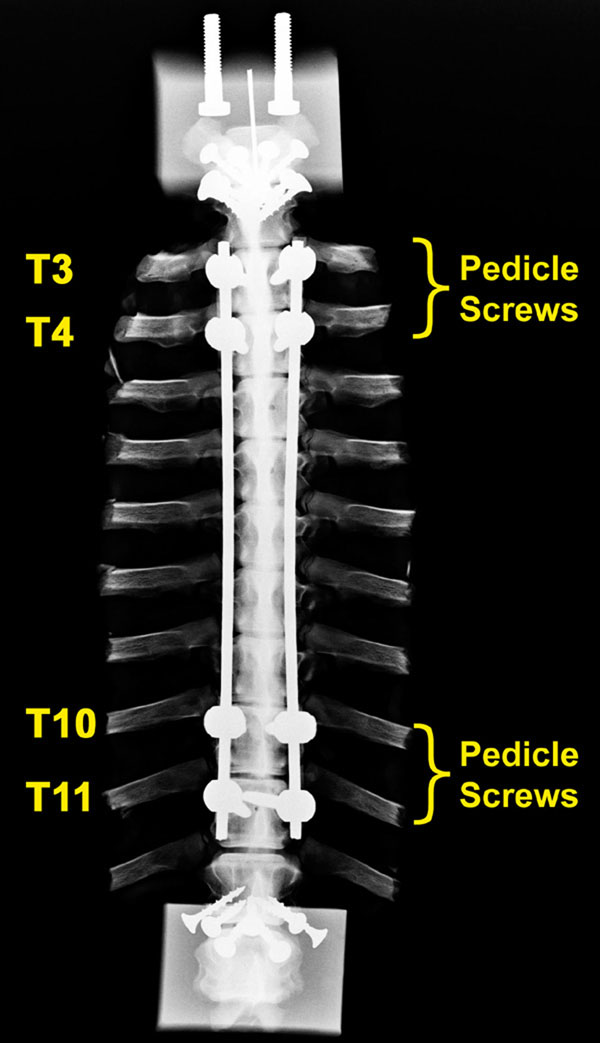
**Radiograph of spine test specimen** Spine test specimen with dual titanium rod construct, coronal view. The rods are anchored using two pairs of pedicle screws at the proximal end, and another two pairs at the distal end. One noninstrumented adjacent disc is above and below the instrumented region.

**Figure 2:**
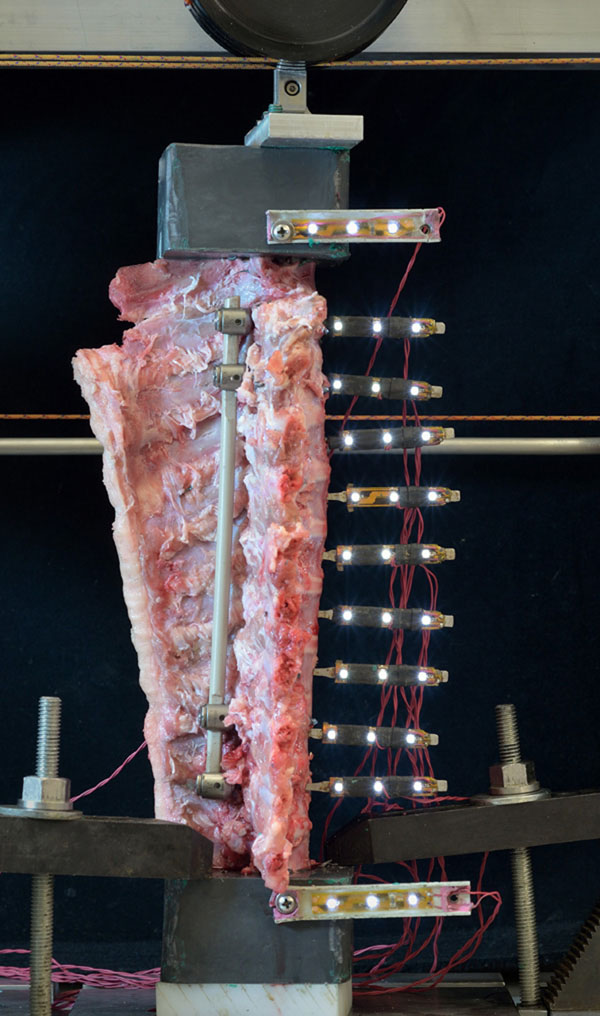
**Photograph of spine test specimen** Spine test specimen with dual PEEK rods, with one rod visible, mounted for flexion-extension test. At each vertebra, a marker array with 3 white LEDs was inserted into the anterior aspect for video motion analysis.

**Figure 3:**
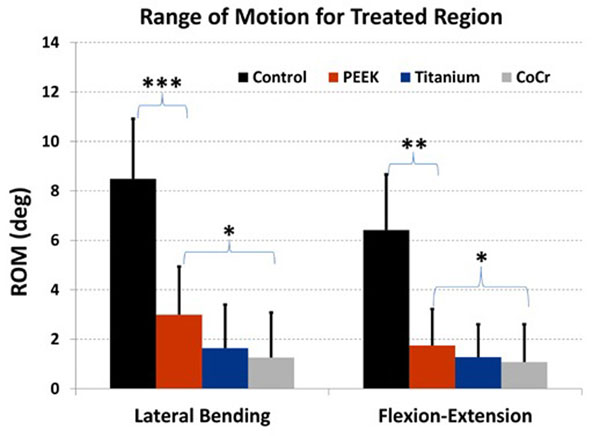
**Range of motion (ROM) over the instrumented region of the spine for four test conditions** Range of motion for control, non-instrumented, spine segments, and for spines instrumented with PEEK, titanium, and cobalt-chrome (CoCr) rods. *** p< 0.0001, ** p< 0.001, * p< 0.01.

Spines were sectioned to include vertebrae T1-T13. Specimens were tested before and after instrumentation. Paired pedicle screws were inserted into T3 and T4 for the proximal anchor, and into T10 and T11 for the distal anchor (Figure 1). An open intervertebral joint remained above and below the surgical construct. Specimens were tested 1) before dual rod insertion, followed by 2) PEEK rods (6.25 mm dia, n=6) (Figure 2), 3) Ti rods (4 mm dia, n=6), or 4) CoCr rods (5 mm dia, n=4). Tests were conducted in lateral bending (LB) and flexion-extension (FE) by applying continuous loads to peak moments exceeding ±5 Nm using a materials test system with cable-pulley attachments. Five cycles were applied, with the fourth analyzed. Vertebral positions at each level were measured from arrays of markers (LEDs) using high definition video. Rotations were calculated using a customized program (MATLAB). Range of motion was defined as the maximum side-to-side rotation for each level. ROM for the treated region was determined by adding ROMs at each instrumented level (T3-T11). Differences in ROM by treatment were determined by two-tailed paired t-tests and Bonferroni correction based on four primary comparisons: PEEK vs control and PEEK vs CoCr, in LB and FE (α = 0.05/4 = 0.0125).

## Results

### Lateral bending

In lateral bending, ROM after each treatment was lower than noninstrumented control, including treatment with PEEK rods, at each of the instrumented levels. PEEK rods allowed greater ROM than both Ti or CoCr rods at every instrumented level. ROM was greater at the proximal and distal noninstrumented segments of instrumented specimens compared to control. Mean ROM at proximal and distal noninstrumented levels was lower for PEEK than for Ti and CoCr. Combining instrumented levels, ROM for spines with PEEK rods was 35% of noninstrumented controls (p<0.0001), and 2.7 x greater than spines with CoCr rods (p<0.01).

### Flexion-extension

In flexion-extension, ROM after each treatment was lower than noninstrumented control, including treatment with PEEK rods, at each of the instrumented levels. PEEK rods usually allowed greater ROM than Ti or CoCr rods at individual levels, but variability was greater in FE than in LB. Mean ROM at proximal and distal noninstrumented levels was lower for PEEK than for Ti and CoCr. Combining instrumented levels (Figure 3), ROM for spines with PEEK rods was 27% of noninstrumented control (p<0.001), and 1.8x greater than spines with CoCr rods (p<0.01).

## Discussion

The large reduction in ROM after instrumentation with PEEK rods indicated significantly increased spine stability due to the construct. Flexible polymeric growing rod constructs significantly decreased range of motion compared to noninstrumented controls. ROM with PEEK rods remained significantly greater than ROM with cobalt-chrome alloy rods. The hypothesis that PEEK rod constructs would clearly not provide sufficient stability for growing rod constructs was not supported. The hypothesis that PEEK rods would provide increased flexibility compared with metal rods was supported. Further, smaller increases in ROM at proximal and distal adjacent discs occurred with PEEK compared to the metal rods, which may decrease propensity for junctional kyphosis [4].

The concept of flexible growing rods has been explored with computational modeling [5]. To the investigators’ knowledge, this is the first biomechanical study of flexible growing rods. Many other studies are necessary to determine the clinical potential of this concept prior to translation. Limitations include in vitro tests on physiologically normal quadruped spines, and intact rods that did not contain any distraction mechanism. Further tests are needed in buckling and torsion, and strength and fatigue properties are essential. Physiological loads of body weight, activity, and curve correction are not yet well defined. The next stages of this preliminary work will likely investigate the effect of a realistic distraction mechanism. Molding of the initial rod configuration into curves to better approximate a desirable sagittal profile may be incorporated. However, results of this early feasibility study indicate that continued investigation into the concept and potential application of flexible growing rods for early onset scoliosis remains warranted.

## Conclusions

In this pilot study, flexible polymeric growing rod constructs decreased range of motion compared to noninstrumented controls, and significantly increased ROM compared to CoCr rod constructs. Growing rods of more flexible materials may eventually help improve treatment options for young patients with severe spinal deformity. Retention of more spine flexibility would likely allow for fewer complications, and higher satisfaction for patients, parents, and caregivers.

This is the extended abstract of IRSSD 2014 program book [6].

## Competing interests

None of the co-authors has a financial disclosure related to this work.

## Authors' contributions

DBA oversaw and participated in the design of the study and methods, performed the statistical analysis, and drafted the manuscript. DLG drafted test methods, designed and fabricated the continuous loading fixture, and oversaw testing. ACB performed the biomechanical tests and carried out the analysis. MFC performed the surgical instrumentation of the specimens, participated in test development, and provided surgical expertise and judgment. MTC helped perform the tests, adapted the program for data analysis, and oversaw the reduction of the data. PFS was responsible for the overall concept and clinical relevance, participated in study design, and reviewed the manuscript.

## Declarations

Publication charges for this collection were funded by IRSSD 2014 Sapporo.

This article has been published as part of *Scoliosis* Volume 10 Supplement 2, 2015: Research into Spinal Deformities 9: Short Papers from the IRSSD 2014 Meeting. The full contents of the supplement are available online at http://www.scoliosisjournal.com/supplements/10/S2.

## References

[B1] Akbarnia BA, Yazici M, Thompson GH The Growing Spine 2011 Heidelberg: Springer

[B2] Ponnappan RK Serhan H Zarda B Patel R Albert T Vaccaro AR Biomechanical evaluation and comparison of polyetheretherketone rod system to traditional titanium rod fixation The Spine Journal 2009 9 263 267 10.1016/j.spinee.2008.08.002 18838341

[B3] Agarwal AK Kodigudla M Desai DS Agarwal A Palepu V Goel VK PEEK rod and Ti screw fixation provides construct stability similar to Ti rod and screw system Transactions Orthopaedic Research Society 2013 808

[B4] Thawrani DP Glos DL Coombs MT Bylski-Austrow DI Sturm PF Transverse process hooks at upper instrumented vertebra provide more gradual motion transition than pedicle screws Spine 2014 39 14 E826 E832 10.1097/BRS.0000000000000367 24732851

[B5] Agarwal A Agarwal AK Jayaswal A Goel VK Effect of distraction force on growth and biomechanics of the spine: A finite element study on normal juvenile spine with growth rod instrumentation Spine Deformity 2014 2 260 269 10.1016/j.jspd.2014.03.007 27927346

[B6] Bylski-Austrow DI Glos DL Bonifas AC Carvalho MF Coombs MT Sturm PF Flexible Growing Rods: Polymer Rods Provide Stability to Skeletally Immature Spines Scoliosis 2015 10 Suppl 1 O73 10.1186/1748-7161-10-S2-S16 PMC4331734 25810752

